# The effects of flipped classroom model on knowledge, behaviour and health beliefs on testicular cancer and self-examination: a randomized controlled trial study

**DOI:** 10.1093/her/cyad007

**Published:** 2023-02-06

**Authors:** Kamil Akcali, Sevinc Tastan

**Affiliations:** Health Sciences Faculty, Nursing Department, Eastern Mediterranean University, Via Mersin 10, Famagusta, North Cyprus 99628, Turkey; Health Sciences Faculty, Nursing Department, Eastern Mediterranean University, Via Mersin 10, Famagusta, North Cyprus 99628, Turkey

## Abstract

This randomized controlled trial study aims to examine the flipped classroom model's effects on the knowledge and health beliefs of testicular cancer and testicular self-examination. The study was conducted on 66 students in Northern Cyprus. A descriptive information form, Champion’s Health Belief Model Scale, visual analogue scale of satisfaction with the learning method, the knowledge questionnaire on testicular cancer and the testicular self-examination itself were used for data collection. The mean posttest knowledge score of the intervention group (14.44) was higher than the mean posttest knowledge score of the control group (12.65). The difference between groups was statistically significant (*P* < 0.05). The mean posttest scores obtained by the intervention group from the barriers and the severity subscales of the Champion’s Health Belief Model Scale were lower than for the control group (*P* < 0.05). The percentages of participants performing the testicular self-examination after receiving training were 82.4% and 59.4% for the intervention group and control group, respectively. The complete trial protocol can be accessed at ClinicalTrials.gov (NCT04851197). Since the flipped classroom model increased the rate of students performing testicular self-exams and the levels of knowledge and satisfaction of the students, the use of this model in different courses may be promoted.

## Introduction

Testicular cancer (TC) accounts for 1.0–1.5% of all malignant cancers, and the incidence of the disease is higher in males aged 15–35 years [1]. In recent decades, the annual incidence of TC has increased 1.80-fold from 37.231 in 1990 to 66.833 in 2016 [2]. According to Turkey Cancer Statistics, the most common cancer in men aged 15–24 is TC with a rate of 26.1% [3]. Although both genetic and environmental factors play a role in the development of TC, the primary risk factor is cryptorchidism [4]. A lump or enlargement in either testicle is the most frequent symptom of TC.

Although the TC cells spread rapidly, the 5-year survival rates of the patients may increase up to 95% if diagnosed early [4]. The prognosis of TC is directly related to early diagnosis [5]. If there is a history of TC in the family, then all male family members are recommended to perform testicular self-examination (TSE) regularly [6, 7]. TSE can be easily performed since it is a safe, economic, easy-to-learn and non-invasive diagnosis method that does not require much time. Regular TSE not only fosters the early detection of TC but also assists young men in making health-related decisions and becoming comfortable with their bodies [6]. When men learn and apply TSE with a good education, they will take more responsibility for their own health [8]. Although there is no recommendation in the national guidelines regarding the use of TSE in the early diagnosis of TC in Turkey, recommendations vary in various countries around the world. The US Preventive Services Task Force does not recommend screening for TC in asymptomatic adolescent or adult males (Grade D recommendation) [9]. The European Association of Urology, on the other hand, emphasizes the importance of self-examination, especially in the presence of clinical risk factors, including a family history of TC, in the general population [5]. However, the existing studies on adolescents and young men have shown that the level of knowledge of TC and regular TSE performance is not adequate [10].

Different psychosocial models explain the attitudes and beliefs influencing health. The Health Belief Model (HBM) is an effective guide to explaining and measuring the factors motivating and preventing early diagnosis behaviours or participating in cancer screening programmes. According to the HBM, revealing the health beliefs of individuals will be beneficial for determining the problem domains before education so that positive health behaviours may be improved [11]. Education programmes on TC and TSE aim to increase individual awareness and promote healthy behaviours on the early diagnosis. There are various education methods and techniques to improve awareness of TSE. In recent years, the flipped classroom model (FCM), which blends different learning methods, has been one of the most dynamic emerging technologies in education [12]. The FCM is a blended learning model that aims to facilitate learning by integrating face-to-face learning in the class through group discussion and problem-solving and distance learning through asynchronous video lessons and online collaboration [13]. Recently, the model has emerged as an alternative to conventional methods in nursing education. The FCM has the potential to motivate nursing students in ways that address the needs of today’s students and the complexity of contemporary health care. The intensity and complexity of current health services find its reflection in the demands for educational reform in healthcare programmes, including nursing, and for new models that will improve the problem-solving, reasoning and decision-making skills of the students [12].

The existing studies of TC and TSE education have shown that videos and classical presentations are being used in education [14]. Contrary to these studies, we will evaluate the effects of the flipped classroom on knowledge and health beliefs on TC and TSE in male nursing students. The findings of this study may guide health professionals to select the most effective learning methods for the early diagnosis of TC and increase the effectiveness of educational activities. This study aimed to examine the effects of the flipped classroom method on knowledge, behaviour and health beliefs on TC and TSE in male nursing students.

## Method

### Study design and setting

This randomized controlled trial was conducted at the department of nursing of a university in Northern Cyprus during the fall semester of the 2020–2021 academic year. In this study, the researcher followed the randomized controlled studies using the Consolidated Standards of Reporting Trials 2015 control list.

### Sampling

G-Power 3.1.9.2 was used to calculate the sample size. A total of 42 participants, 21 participants in each of the two groups, were required for a 95% confidence interval, 80% power and 0.8 effect size. In this study, Cohen’s d effect size was used to calculate the effect size of two independent groups [15]. Due to possible withdrawals from the study, 68 volunteer male nursing students aged 18 years and over, who had not received any prior education in TSE, were included in the study. The curriculum of the nursing school where the study was conducted consists of eight semesters over four academic years. The first-year curriculum includes non-nursing courses. Nursing courses are given from the second year onwards. For this reason, the sample group of the study consisted of first-year male students who had not received any previous training in TSE.

### Randomization and blinding

**Fig. 1.**
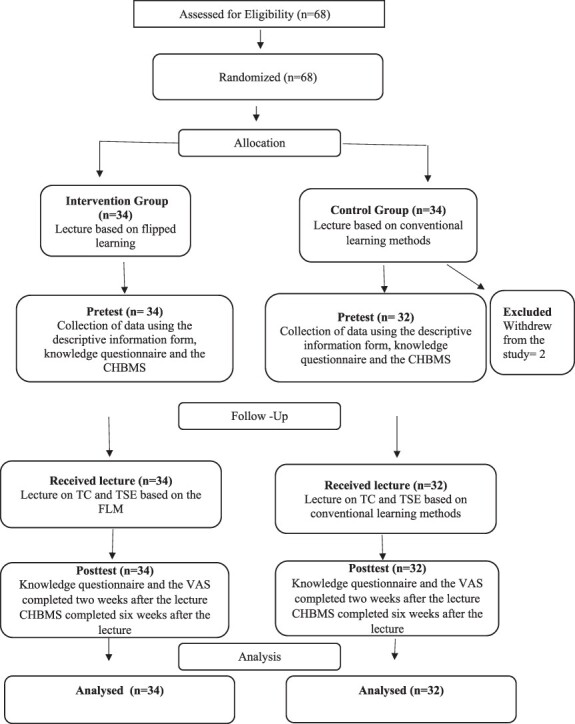
The Consolidated Standards of Reporting Trials (CONSORT) 2010 flow diagram of the participants through each stage of the study.

A simple randomization method was used to allocate the participants into two groups with equal numbers. The intervention group (*n* = 34) received a lecture using the FCM, whereas the control group (*n* = 34) received a traditional lecture. Since two students in the control group did not want to continue, the study was finalized with 66 students, including 34 and 32 students in the intervention group and the control group, respectively (Fig. 1). The collected data were analysed by a researcher, who did not take part in giving the lecture, and the titles of the groups were masked during the data analysis. This study was conducted online during the Covid-19 pandemıa lockdown period. Our students represented different countries in addition to Northern Cyprus. In the online education, separate classes were created for the control and intervention groups in the Microsoft Teams software program. To avoid the interaction between the groups, the training was given to the control and intervention groups, one after the other, on the same day.

### Data collection tools

A descriptive information form, a knowledge questionnaire on TC and TSE and the Champion’s Health Belief Model Scale (CHBMS) were used for data collection.

#### Descriptive information form

This form consisted of 11 questions, including 2 questions on sociodemographic characteristics (age and marital status) and 9 questions on the characteristics of TC and TSE, such as individual and family history, perceived TC risk, level and source of knowledge, regular TSE performance and the reasons for not performing TSE [16, 17]. A visual analogue scale (VAS) was also included in the descriptive form to measure satisfaction with the learning method after the lecture.

#### Knowledge questionnaire on TC and TSE

This questionnaire contained 16 questions on the frequency and risk factors of TC, the importance of the early diagnosis of TSE and the frequency and method of TSE. The participants were asked to respond to each question on a three-point Likert scale, with the possible responses being ‘yes’ (1 point), ‘no’ (0 points) and ‘don’t know’ (0 points). Possible scores ranged between 0 and 16, with higher scores indicating a higher level of knowledge. Expert opinion was received from nine nursing academicians from the field to achieve content validity [18]. Two of these nurse academicians had clinical nursing experience in the urology service. Others had specialties in surgical nursing. An email asking the experts to evaluate the knowledge questionnaire and the educational video was sent to the experts. The content validity index values of the questionnaire (96.3%) and the video (94.5%) were higher than 0.80.

#### CHBMS

The CHBMS was developed by Barnes (2000) and translated into Turkish by Pınar *et al*. (2011) to identify the knowledge and practice of TSE. The scale consists of 26 items in 5 subscales, namely susceptibility (items 1–5), severity (items 6–12), benefits (items 13–15), barriers (items 16–20) and self-efficacy (items 21–26). Cronbach’s alpha of the Turkish version of the scale was 0.91, whereas Cronbach’s alpha of the subscales in our study ranged from 0.64 to 0.92. The permission to use the Turkish version of the scale was obtained via email.

### Interventions

Data were collected online from December 2020 to March 2021 via Google Forms survey software. Due to the Covid-19 pandemic at the time of the study, all training sessions were conducted online in our country, so this study was carried out entirely online. After obtaining the necessary permissions, the participants were allocated to the intervention group or the control group randomly. In the Microsoft Teams program, separate classes were created for each of the two groups. In this study, the FCM was used for the students in the intervention group, whereas a traditional PowerPoint presentation was used for the control group. This was the first time that a FCM was used in the nursing programme in which the study was conducted. Before the lectures, participants in both groups were asked to complete the descriptive information form, the knowledge questionnaire on TC and TSE and the CHBMS uploaded to Google Forms. Next, for the intervention group, the educational materials, including the PowerPoint presentation and asynchronous videos, were uploaded onto the Microsoft Teams program, and the students were asked to study these educational materials before the weekly lecture. The content of the PowerPoint presentation and the prepared training video included the anatomical structure of the testicles, the definition of TC, its incidence, TC risk factors, TC symptoms, diagnostic methods, the importance of TSE and how to do it. For the intervention group, the students then came to the training session, which was designed according to the FCM, including a question-and-answer session, and had no classical lecture presentation. The training was given only once and lasted 40 min. Two weeks after the course, the students in the intervention group were asked to complete the knowledge questionnaire and the VAS on satisfaction as a posttest. Six weeks after the course, the intervention group completed the CHBMS as a posttest.

The students in the control group, on the other hand, had a different learning path. They received a 30-min PowerPoint presentation during the lecture, and in the last 10 min of the session, they answered questions on the material they received. The PowerPoint presentation used for the control group was the same as the training material for the intervention group. Two weeks later, the students in the control group completed the knowledge questionnaire and the VAS. Six weeks after the course, the control group also completed the CHBMS as a posttest.

To avoid the interaction between the groups, the training was given to the control and intervention groups, in quick succession, on the same day. The training was given once for both groups. The complete trial protocol can be accessed at ClinicalTrials.gov (NCT04851197).

### Outcomes

The knowledge questionnaire on TC and TSE and the CHBMS were used to evaluate the knowledge and health awareness concerning TC and TSE amongst male nursing students. The VAS was used to evaluate student satisfaction with the FCM.

### Statistical analysis

The collected data were analysed using the Statistical Package for Social Sciences (SPSS) version 24.0 (IBM, Armonk, NY, USA). Descriptive characteristics of the participants were presented by the frequency of the responses. Descriptive statistics, including mean, standard deviation and minimum and maximum values, were used to present the scores obtained from the CHBMS and the knowledge questionnaire. The Kolmogorov–Smirnov test was used to examine the suitability of the data for normal distribution. Pearson’s chi-squared test and Fisher’s exact test were used to compare descriptive characteristics. The Mann–Whitney U test was used to compare the scores obtained from the knowledge questionnaire, and the VAS judged student satisfaction with the learning experience. The paired sample *t*-test was used for intragroup comparison, whereas an independent-sample *t*-test was used for intergroup comparison.

### Ethical considerations

Permission was obtained from the Scientific Research and Publication Ethics Committee (ETK00-2020-0279) and the institution to conduct the study. Written informed consent was obtained from all participants.

## Results

The comparison of some of the descriptive characteristics of the two groups can be seen in Table I. The mean age of the participants was 20.71 ± 1.11 years. In addition, 94.1% of the participants in the intervention group and 93.8% in the control group did not have testicular problems. The percentages of participants who did not regularly perform TSE in the intervention group and the control group were 97.1% and 90.6%, respectively. On the other hand, 70.6% of the participants in the intervention group and 56.3% in the control group had no previous knowledge of TC. There was no statistically significant difference between the two groups in terms of age, testicular problems, perceived TC risk, level of knowledge, regular TSE performance and the reasons for not performing TSE (*P* > 0.05).

**Table I.** Intergroup comparison of the descriptive characteristics

	Intervention (*n* = 34)		Control (*n* = 32)		Total (*n* = 78)			
	Mean	SD	Mean	SD	Mean	SD	Test	*P*
Age	20.88	1.17	20.53	1.01	20.71	1.11	−1.295	0.200
	*N*	%	*n*	%	*n*	%	X^2^	*P*
Testicular problems								
No	32	94.1	30	93.8	62	93.9	–	1.00^a^
Yes	2	5.9	2	6.2	4	6.1		
Family history of TC								
No	34	100	32	100	66	100	–	–
Risk perception								
No risk	25	73.5	27	84.4	52	78.8	0.281	0.371
Normal	9	26.5	5	15.6	14	21.2		
Regular TSE performance								
No	33	97.1	29	90.6	62	92.4		0.348^a^
Yes	1	2.9	3	9.4	4	6.1		
Reasons for not performing TSE								
Do not know	29	87.9	23	79.3	50	83.3	0.838	0.658
Do not pay attention	2	6.1	3	10.3	5	8.3		
Fear of detecting TC	2	6.1	3	10.3	5	8.3		
Has knowledge								
No	24	70.6	18	56.3	42	63.6	0.226	0.307
Yes	10	29.4	14	43.7	24	36.4		

a Fisher’s exact test.

Next, the comparison of the scores obtained from the knowledge questionnaire and the subscales of the CHBMS is presented in Table II. The mean pretest scores obtained by the intervention group (6.09 ± 4.75) and the control group (7.18 ± 3.89) on the knowledge questionnaire were not statistically significantly different (*P* > 0.05). Similarly, the scores obtained by the participants in both groups from the subscales of the CHBMS were not statistically significant (*P* > 0.05).

**Table II.** Pretest scores obtained from the knowledge questionnaire and the CHBMS

	Possible min–max scores	Mean (SD)	Test	*P*
Knowledge questionnaire				
Intervention group	(0–16)	6.09 ± 4.75	−1.120^a^	0.263
Control group	(0–16)	7.18 ± 3.89		
Susceptibility				
Intervention group	(5–25)	10.68 ± 3.98	−0.608^b^	0.546
Control group	(5–25)	10.13 ± 3.34		
Severity				
Intervention group	(5–35)	21.50 ± 6.14	0.021^b^	0.983
Control group	(5–35)	21.53 ± 5.92		
Benefits				
Intervention group	(5–15)	10.44 ± 3.07	−0.570^b^	0.571
Control group	(5–15)	10.03 ± 2.75		
Barriers				
Intervention group	(5–25)	10.65 ± 3.39	0.737^b^	0.464
Control group	(5–25)	11.25 ± 3.24		
Self-efficacy				
Intervention group	(5–30)	17.03 ± 4.65	−0.075^b^	0.940
Control group	(5–30)	16.94 ± 5.29		

a Mann–Whitney U test.

b Student’s *t*-test.

Table III presents a comparison of the scores obtained from the knowledge questionnaire, the VAS on satisfaction with the learning method and the subscales of the CHBMS. Accordingly, the mean score obtained by the intervention group (14.44 ± 1.84) from the knowledge questionnaire was statistically significantly higher than that of the control group (12.65 ± 3.89) (*P* < 0.05). Moreover, the mean posttest scores obtained by the intervention group from the severity (1906 ± 5.07) and the barriers (9.06 ± 3.04) subscales of the CHBMS were statistically significantly lower than the scores obtained by the control group (21.81 ± 5.45; 11.06 ± 3.99, respectively) (*P* < 0.05). Furthermore, the mean score obtained from the VAS by the intervention group (9.62 ± 0.73) was statistically significantly higher than the score of the control group (8.66 ± 1.72) (*P* = 0.007). Finally, 82.4% of the participants in the intervention group and 59.4% in the control group performed TSE after the lectures, and the difference between the groups was statistically significant (*P* = 0.039). The FCM yielded a much increased rate of students performing testicular self-exams.

**Table III.** Posttest scores obtained from the knowledge questionnaire, CHBMS and the VAS on satisfaction

	Intervention,	Control,		
	Mean ± SD	Mean ± SD	Test	*P*
Knowledge questionnaire	14.44 ± 1.84	12.65 ± 3.89	−2.165^a^	0.030
CHBMS				
Susceptibility	10.74 ± 3.02	10.97 ± 3.25	0.303^b^	0.763
Severity	19.06 ± 5.07	21.81 ± 5.45	2.126^b^	0.037
Benefits	12.50 ± 2.22	11.75 ± 3.09	−1.138^b^	0.259
Barriers	9.06 ± 3.04	11.06 ± 3.99	2.298^b^	0.025
Self-efficacy	23.59 ± 4.36	21.50 ± 4.87	−1.836^b^	0.071
VAS on satisfaction	9.62 ± 0.73	8.66 ± 1.72	−2.677^a^	0.007
	*N* (%)	*N* (%)	Test	*P*
Regular TSE performance				
No	6 (17.6)	13 (40.6)	–	0.039^c^
Yes	28 (82.4)	19 (59.4)		

a Mann–Whitney U test.

b Independent Student *t*-test.

c Pearson’s chi-squared test.

## Discussion

Despite high cure rates, TC continues to pose a serious threat, especially to young adult men. Testicular self-exam is the primary health-protective behaviour in the early diagnosis and treatment of TC. In this study, the majority of the participants did not know about TC and did not perform TSE regularly prior to the intervention. In the existing studies, the lack of knowledge of TC in university students ranged between 65.6% and 98% [10, 17, 19–21], and 66.6–88.3% of the students did not know how to perform TSE [10, 20, 22, 23]. Another study of final-year medical students in Nigeria found that 76.4% of the participants did not perform TSE regularly [24]. Pietrzyk *et al*. (2020) reported that 30% of the medical students in Poland performed TSE once a month, with the reasons for not performing TSE including a lack of skill and knowledge for performing TSE, as well as a fear of detecting cancer [25]. Since the university students constitute one of the groups with the highest risk of TC, their awareness of TC and TSE, which is reported to be low, should be increased. Also, it is seen that there is a lack of knowledge of the participants in the studies conducted in the society about TC and TSE. A study conducted in the Maltese Islands discovered that 66.1% of men have never done TSE, and 90.2% were ready to do TSE regularly after they were informed of the method of doing one [26]. Research conducted by Alamri *et al*. stated that 61.4% of 809 men aged between 15 and 55 years old had never done TSE, and the awareness of men over 50 years of age is better than that of young adults [27]. On the other hand, Milecki *et al*. found in their study of 522 participants with an average age of 32 years that 57.5% of the participants had never done TSE and only 12% of them did it once a month. The same study emphasized that a family history of TC, a doctor’s recommendation, social campaigns and partners’ concerns are among the factors associated with TSE application [22]. The results of the current study are similar to the literature. Health professionals are in a very good position to provide TSE training to young adult men who come to the health institution. Increasing the awareness of students and the society about TC and TSE and filling the gap in knowledge should be among the priorities of health professionals.

In the current study, participants in the intervention group, which received a lecture using the flipped classroom, obtained higher scores compared with the control group, which received a traditional lecture. The existing studies of receiving a lecture about TC and TSE and its effects on performing TSE reported that the frequency of TSE performance increased after the lecture [14, 17]. Additionally, the participants who received a lecture using the flipped classroom were more satisfied with the course than the students receiving a traditional lecture. Similar to our findings, other studies reported that the use of FCM increased student satisfaction and had positive effects on participants’ knowledge and skills [28–32]. Higher satisfaction with the course in the intervention group of our study relates to the fact that the FCM facilitates learning. Another study that used the FCM to teach a renal pharmacotherapy module also found that the model improved student performance due to contact with the course material before classes, the benchmark and formative assessments administered during the module and the interactive class activities [33]. The FCM is a student-based learning method that prioritizes individual preparations before class and directs the students to problem-solve, reason and apply theory into practice [12]. Based on these findings, we may suggest that the FCM, which is an effective method to improve success and motivation, may be more widely used in nursing education.

This study analysed the effects of courses based on the FCM compared with the conventional methods of knowledge and health beliefs on TC and TSE. The HBM is an important guide for explaining and measuring the factors that improve, motivate or prevent individual health. Mean posttest scores obtained by the participants in the intervention group from the severity and barriers subscales of the CHBMS were significantly lower than those of the control group. Although statistically insignificant, the mean scores obtained by the intervention group from the benefits and the self-efficacy subscales were higher than those by the control group. A study by Pour and Çam, which used PowerPoint presentation, video and question-and-answer techniques to give a lecture on TC and TSE, found that the posttest scores obtained by the intervention group from the subscales of susceptibility and barriers decreased, whereas the scores from the subscale of benefits increased [17]. Another study by Demir *et al.* found that the self-efficacy scores of the participants who regularly performed TSE and had a higher level of knowledge of TC were higher [23]. Gümüş and Terzi reported that the participants with a lower level of knowledge of TSE obtained lower scores from the subscale of barriers and higher scores from the self-efficacy subscale of the CHBMS [34]. Our findings are mostly parallel to the findings in the relevant literature [35–38]. According to Champion and Skinner, there is a positive relationship between TSE performance, susceptibility and the level of caring about TC [39]. One study indicates that men with greater self-efficacy towards testicular self-exams are more likely to feel comfortable doing monthly exams and discussing testicular abnormalities with a healthcare professional [40]. Research-based approaches to education and counselling may improve the susceptibility, severity, self-efficacy and benefits dimensions of the CHBMS, which in turn, may contribute to positive health behaviours [41].

This study has some limitations. One of them is that the results can only be generalized to this group because it was conducted in a single centre. Another limitation is that the sample group was limited to male students only since the effects of the flipped classroom method on the knowledge and health beliefs of TC and TSE, as well as on TSE behaviour, were examined in this study. The last limitation is the inclusion of more than 61% of the sample size due to the concern that there would be too many dropouts from the study because of the Covid-19 pandemic, and all 68 students who met the inclusion criteria volunteered to participate in the study.

## Conclusion

Sustaining educational activities to improve awareness of TC and TSE in university students aged 18–35 years is vital. Although the scores obtained by the groups in our study increased after the course on TC and TSE, participants in the intervention group, who received a lecture based on flipped classroom methods, had a higher level of knowledge afterwards and registered satisfaction with the course. Also, the FCM increased the rate of students performing testicular self-exams. Consequently, the FCM may be used as a student-friendly learning method in nursing education, which may increase student satisfaction, the level of knowledge and regular TSE performance. Further studies on different groups may evaluate the effectiveness of the FCM. Also, this study may be replicated in more than one centre where women will be included to motivate them to actively participate in testicular partner examination.

## References

[1] Le Cornet C, Lortet-Tieulent J, Forman D et al. Testicular cancer incidence to rise by 25% by 2025 in Europe? Model-based predictions in 40 countries using population-based registry data. *Eur J Cancer* 2014; 50: 831–9. 24369860 10.1016/j.ejca.2013.11.035

[2] Wang S-C, Chang N-W, Chen W-J et al. Trends of testicular cancer mortality-to-incidence ratios in relation to health expenditure: An ecological study of 54 countries. *Int J Environ Health Res* 2021; 18: 1546. 10.3390/ijerph18041546 PMC7914754 33561945

[3] General Directorate of Public Health . *Turkey Cancer Statistics 2017*. Ankara: Ministry of Health, 2021.

[4] Gilligan T, Lin DW, Aggarwal R et al. Testicular cancer, version 2.2020, NCCN clinical practice guidelines in oncology. *J Natl Compr Canc Netw* 2019; 17: 1529–54. 31805523 10.6004/jnccn.2019.0058

[5] Laguna MP, Albers P,Algaba F et al. EAU guidelines on testicular cancer. *EurAssoc Urol* 2022: 1–65.

[6] Thornton CP . Best practice in teaching male adolescents and young men to perform testicular self-examinations: A review. *J Pediatr Health Care* 2016; 30: 518–27. 26778347 10.1016/j.pedhc.2015.11.009

[7] Ustundag H . Assessment of the testicular self-examination knowledge and health belief model of health sciences students. *In J Caring Sci* 2019; 12: 972–8.

[8] Fadich A, Giorgianni SJ, Rovito MJ et al. USPSTF testicular examination nomination – Self-examinations and examinations in a clinical setting. *Am J Mens Health* 2018; 12: 1510–6. 29717912 10.1177/1557988318768597 PMC6142159

[9] U.S. Preventive Services Task Force. Screening for testicular cancer: U.S. Preventive Services Task Force reaffirmation recommendation statement. *Ann Intern Med* 2011; 154: 483–6. 21464350 10.7326/0003-4819-154-7-201104050-00006

[10] Seher Y, Saglam R, Kadioglu H. Knowledge, beliefs and practices of university students regarding testicular cancer and testicular self-examination. *Clin Exp Health Sci* 2020; 10: 235–40.

[11] Rosenstock IM . The health belief model and preventive health behavior. *Health Educ Monogr* 1974; 2: 354–86.

[12] Betihavas V, Bridgman H, Kornhaber R et al. The evidence for ‘flipping out’: A systematic review of the flipped classroom in nursing education. *Nurse Educ Today* 2016; 38: 15–21. 26804940 10.1016/j.nedt.2015.12.010

[13] Zainuddin Z, Halili SH. Flipped classroom research and trends from different fields of study. *Int Rev Res Open Distance Learn* 2016; 17: 313–40.

[14] Akar ŞZ, Bebiş H. Evaluation of the effectiveness of testicular cancer and testicular self-examination training for patient care personnel: Intervention study. *Health Educ Res* 2014; 29: 966–76. 25248831 10.1093/her/cyu055

[15] Cohen J . *Statistical Power Analysis for the Behavioral Sciences*. New York: Routledge, 2013.

[16] Iyigun E, Tastan S, Ayhan H et al. Validity and reliability analysis of the planned behavior theory scale related to the testicular self-examination in a Turkish context. *Postgrad Med J* 2016; 128: 496–501. 10.1080/00325481.2016.1182872 27130481

[17] Pour HA, Çam R. Evaluation of men’s knowledge, attitude and behavior about testicular self-examination and testicular cancer. *Florence Nightingale J Nurs* 2014; 22: 33–8.

[18] Davis LL . Instrument review: Getting the most from a panel of experts. *Applied Nursing Research* 1992; 5: 194–7.

[19] Ramim T, Mousavi SQ, Rosatmnia L et al. Student knowledge of testicular cancer and self-examination in a medical sciences university in Iran. *Basic Clin Cancer Res* 2014; 6: 7–11.

[20] Sayar S Erdem M, Göktaş A et al. Examining of university students’ awareness, beliefs and practices about testicular self examination. *KTO Karatay Üniversitesi Saglik Bilimleri Dergisi* 2021; 2: 9–19.

[21] Ilo IJ, Omeye OB, Ede SS et al. Knowledge, attitude and practice of testicular self examination among male undergraduate students of University of Nigeria Enugu Campus. *J Drug Deliv Ther* 2022; 12: 78–85.

[22] Milecki T, Majchrzak N, Balcerek A et al. Attitudes about testicular self-examination among Polish males. *Biology* 2021; 10: 1–9. 10.3390/biology10030239 PMC8003475 33808756

[23] Demir B, Polat HT, Ploutz-Snyder RJ. The effect of testicular cancer and testicular self-examination on knowledge, attitude and health beliefs in university students in Turkey. *J Health Res* 2021; 36: 494–502.

[24] Ugwumba FO, Ekwueme OEC, Okoh AD. Testicular cancer and testicular self-examination; knowledge, attitudes and practice in final year medical students in Nigeria. *Asian Pac J Cancer Prev* 2016; 17: 4999–5003. 28032730 10.22034/APJCP.2016.17.11.4999 PMC5454710

[25] Pietrzyk Ł, Denisow-Pietrzyk M, Czeczelewski M et al. Cancer education matters: A report on testicular cancer knowledge, awareness, and self-examination practice among young Polish men. *Sci Rep* 2020; 10: 1–9. 33244121 10.1038/s41598-020-77734-3 PMC7693263

[26] Marmarà V Aquilina R, Formosa E et al. Testicular cancer awareness among men residing in the Maltese Islands. 2020.

[27] Alamri A, Alqahtani YM, Al-Mudhi MM et al. Knowledge, attitudes and practice toward testicular cancer and testicular self-examination among adolescents and young adults in Aseer region, Saudi Arabia. *Middle East J Fam Med* 2021; 19: 75–85.

[28] Kim HS, Kim MY, Cho M-K et al. Effectiveness of applying flipped learning to clinical nursing practicums for nursing students in Korea: A randomized controlled trial. *Int J Nurs Pract* 2017; 23: 1–10. 10.1111/ijn.12574 28741796

[29] Hanson J . Surveying the experiences and perceptions of undergraduate nursing students of a flipped classroom approach to increase understanding of drug science and its application to clinical practice. *Nurse Educ Pract* 2016; 16: 79–85. 26494304 10.1016/j.nepr.2015.09.001

[30] Wong TH, Ip EJ, Lopes I et al. Pharmacy students’ performance and perceptions in a flipped teaching pilot on cardiac arrhythmias. *Am J Pharm Educ* 2014; 78: 1–6. 25657372 10.5688/ajpe7810185 PMC4315207

[31] Tavakoli AM, Bahonar E, Rafie F et al. Investigating the relationship between motivational beliefs and self-regulation learning with students’ academic performance. *J Adv Pharm Res| Jan-Mar* 2020; 10: 148–52.

[32] Al-Mugheed K, Bayraktar N. Effectiveness of flipped classroom among nursing students on venous thromboembolism (VTE). *Niger J Clin Pract* 2021; 24: 1463–73. 34657011 10.4103/njcp.njcp_129_20

[33] Pierce R, Fox J. Vodcasts and active-learning exercises in a “flipped classroom” model of a renal pharmacotherapy module. *Am J Pharm Educ* 2012; 76: 1–5. 23275661 10.5688/ajpe7610196 PMC3530058

[34] Gümüş K, Terzi B. Evaluation of individuals’ health beliefs and their association with testicular self-examination: Adult sample from Amasya. *J Res Nurs* 2018; 23: 505–17. 34394466 10.1177/1744987118791337 PMC7932397

[35] El Mezayen SE, Abd El-Hay SA. Effect of educational guidelines based on health belief model regarding testicular cancer knowledge, practice and beliefs among male nursing students. *Clin Nurs Stud* 2019; 7: 27–41.

[36] Jasim NA, Naji AB. Using the constructs of the health belief model in changing the health beliefs of male nurses about testicular self-examinations. *Indian J Public Health Res Dev* 2018; 9: 1252–7.

[37] Khani Jeihooni A, Jormand H, Ansari M et al. The effect of educational intervention based on health belief model and social support on testicular self-examination in sample of Iranian men. *BMC Cancer* 2021; 21: 1–10. 34112094 10.1186/s12885-021-08411-5 PMC8194024

[38] Sagir FN, Altinel B. Effects of information provided to university students through an educational brochure on health beliefs and testicular self-examination. *J Cancer Educ* 2022; 1–7. 10.1007/s13187-022-02166-8 35486360

[39] Champion VL, Skinner CS. The health belief model. *Health Educ Behav* 2008; 4: 45–65.

[40] Roy RK, Casson K. Attitudes toward testicular cancer and self-examination among Northern Irish males. *Am J Mens Health* 2017; 11: 253–61. 27645516 10.1177/1557988316668131 PMC5675290

[41] Pınar G, Öksüz E, Beder A et al. Testis kanseri taramalarında Champion’un sağlık inanç modeli ölçeğinin Türkçe uyarlamasının güvenirlik ve geçerliliği. *Tip Arastirmalari Dergisi* 2011; 9: 89–96.

